# Pandemic spotlight on urban water quality

**DOI:** 10.1186/s13717-020-00231-y

**Published:** 2020-05-06

**Authors:** Dennis W. Hallema, François-Nicolas Robinne, Steven G. McNulty

**Affiliations:** 1 Department of Forestry and Environmental Resources, North Carolina State University, 3041 East Cornwallis Road, Research Triangle Park, NC 27709 USA; 2 Canadian Partnership for Wildland Fire Science, Department of Renewable Resources, University of Alberta, 751 General Services Building, Edmonton, AB T6G 2H1 Canada; 3 Eastern Forest Environmental Threat Assessment Center, Southern Research Station, U.S. Department of Agriculture Forest Service, Research Triangle Park, NC 27709 USA

## Abstract

Surface water improvements associated with the COVID-19 economic slowdown illustrate environmental resiliency and societal control over urban water quality.

## Correspondence

Reliable urban water supplies are essential to economic activity and depend on the continuous availability of clean water delivered by rivers (Haddeland et al. 2014). Estimates indicate that urban water demand may increase up to 80% by 2050 (Flörke et al. 2018); however, the ability to meet the increasing demand varies locally. Critical factors are the level of accessibility (Livesley et al. 2016), water treatment infrastructure (Krueger et al. 2019), and impacts from recurring summer drought, wildfires (Hallema et al. 2018), and urbanization (Livesley et al. 2016). Recent observations of rapid environmental recovery add a new dimension to the debate, namely that of the potential impacts of COVID-19 pandemic responses on surface water quality.

**Fig. 1:**
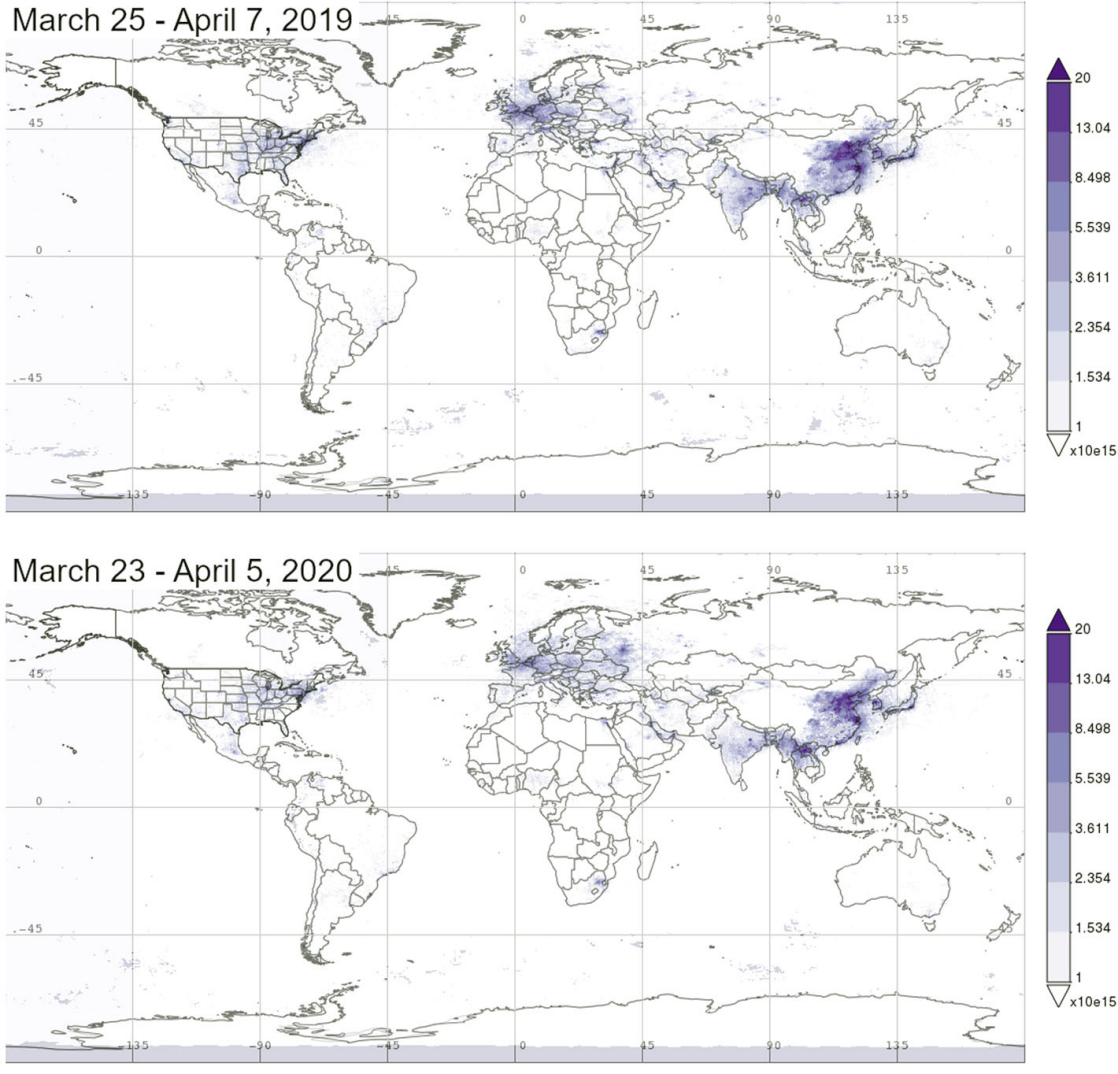
NO_2_ (nitrogen dioxide) in the tropospheric column (mol cm^−2^) for the first 14 days of spring 2019 (top) and 2020 (bottom), respectively, several weeks after many countries worldwide initiated lockdowns [maps generated with the Giovanni online tool (Prados et al. 2010) using data from the Ozone Monitoring Instrument on NASA’s Aura satellite, 30% cloud screened, daily 0.25° resolution]

Most poignant however are the improvements in air quality. Satellite data are showing significant reductions of NO_2_ (nitrogen dioxide) over major cities in China, Europe, the USA, and India following lockdowns enforced to slow the spread of SARS-CoV-2 (NASA Earth Observatory 2020) (Fig. 1). In Venice, Italy, canal water appeared visibly clearer following the lockdown (but not necessarily cleaner). Presumably, reduced boat traffic on the canals resulted in lower sediment concentrations near the water’s surface (Link 2020).

Notwithstanding the disastrous effects on public health and economic welfare, these cases are remarkable illustrations of the highly dynamic nature of environmental systems driven by natural processes like the wind and tides. As post-pandemic water quality data becomes available, we can also expect to see examples of temporarily improved surface water quality in urban areas. The nature and extent of these improvements will depend on the level of urbanization (regional and distribution within the watershed), climate (predominant wind direction and precipitation), and physical characteristics of the watershed (topography and infiltration dynamics of the soil). The respective contributions of groundwater and storm drainage to river flow can indicate how reduced input of contaminants affects surface water quality. Because upstream portions of watersheds often have a lower level of human disturbance, this is where large basins tend to exhibit a higher level of resilience (Hallema et al. 2019).

Under an economic slowdown scenario, headwaters hundreds of kilometers away from urban areas will experience reduced deposition from nonpoint sources of pollution (regionally produced nitrogen dioxide, sulfur dioxide, ammonia, and other gaseous pollutants). For example, given the reduction in nitrogen dioxide concentrations observed over Eastern China, Europe, the Northeastern United States, and India (Fig. 1), this is where urban surface water quality may start showing signs of temporary recovery. Downstream surface waters may also receive less input from both point sources of pollution (e.g., industrial sites) and nonpoint sources (e.g., motorized traffic), as the lockdowns continue. Under an economic worst-case scenario, input from other major nonpoint sources, like mining and agriculture, can potentially decline due to ripple effects created by a slowdown of the global economy. The effects of reduced point source inputs on surface water quality might become evident within a matter of months while the effect of reduced nonpoint source inputs could take much longer to measure.

Given the frequent occurrence of urban water crises, it is critical to document how COVID-19 pandemic response management affects natural processes and surface water quality in the short term. It is equally important to determine how we can better optimize the natural function of water supply areas (Sun et al. 2018) once economic recovery is underway. The current challenge, though, is that economic sectors and industries that contribute to pollution (e.g., energy, consumer, pharmaceutical, and other industries) receive little incentive to promote urban water quality beyond what the law requires. They are chiefly evaluated in terms of total returns to the shareholders; however, an important collateral benefit of economic performance is the creation of jobs and public wealth (i.e., taxation revenue). To improve the sustainability of water supplies and cover the associated cost, it will be necessary for the public, by way of governance, to improve laws and direct public wealth toward outcomes that simultaneously support environmental and economic goals (Claassen et al. 2018).

There are very few positive aspects to be derived from the current public health emergency. The environmental responses to the economic slowdown emphasize in the first place the negative effect of humans on their environment. However, they are also a reminder that society has the ability to improve urban water quality in normal times, provided we make a conscious decision and concerted effort to create the conditions needed to reach that goal.

## Data Availability

Data are publicly accessible via NASA Earthdata’s Giovanni web interface, https://giovanni.gsfc.nasa.gov/.

## Abbreviations

COVID-19: Coronavirus disease 2019
NASA: National Aeronautics and Space Administration
SARS-CoV-2: Severe acute respiratory syndrome coronavirus 2
